# Author Correction: The effect of thermal photons on exceptional points in coupled resonators

**DOI:** 10.1038/s41598-023-43997-9

**Published:** 2023-10-06

**Authors:** Grzegorz Chimczak, Anna Kowalewska-Kudłaszyk, Ewelina Lange, Karol Bartkiewicz, Jan Peřina

**Affiliations:** 1 Institute of Spintronics and Quantum Information, Faculty of Physics, Adam Mickiewicz University, 61-614 Poznań, Poland; 2 RCPTM, Joint Laboratory of Optics of Palacký University and Institute of Physics of Czech Academy of Sciences, 17. Listopadu 12, 771 46 Olomouc, Czech Republic

Correction to: *Scientific Reports* 10.1038/s41598-023-32864-2 published online 11 April 2023

In the original version of this Article, Jan Peřina Jr. was incorrectly affiliated with ‘Institute of Spintronics and Quantum Information, Faculty of Physics, Adam Mickiewicz University, 61‑614 Poznań, Poland.’

The correct affiliation is as follows:

RCPTM, Joint Laboratory of Optics of Palacký University and Institute of Physics of Czech Academy of Sciences, 17. Listopadu 12, 771 46 Olomouc, Czech Republic

The original Article has been corrected.

